# DNA methylation and substance-use risk: a prospective, genome-wide study spanning gestation to adolescence

**DOI:** 10.1038/tp.2016.247

**Published:** 2016-12-06

**Authors:** C A M Cecil, E Walton, R G Smith, E Viding, E J McCrory, C L Relton, M Suderman, J-B Pingault, W McArdle, T R Gaunt, J Mill, E D Barker

**Affiliations:** 1 Department of Psychology, Institute of Psychiatry, Psychology & Neuroscience, King's College London, London, UK; 2 Department of Psychology, Georgia State University, Atlanta, GA, USA; 3 Exeter Medical School, Exeter University, Exeter, UK; 4 Division of Psychology and Language Sciences, Department of Clinical, Educational and Health Psychology, University College London, London, UK; 5 School of Social and Community Medicine, University of Bristol, Bristol, UK

## Abstract

Epigenetic processes have been implicated in addiction; yet, it remains unclear whether these represent a risk factor and/or a consequence of substance use. Here, we believe we conducted the first genome-wide, longitudinal study to investigate whether DNA methylation patterns in early life prospectively associate with substance use in adolescence. The sample comprised of 244 youth (51% female) from the Avon Longitudinal Study of Parents and Children (ALSPAC), with repeated assessments of DNA methylation (Illumina 450k array; cord blood at birth, whole blood at age 7) and substance use (tobacco, alcohol and cannabis use; age 14–18). We found that, at birth, epigenetic variation across a tightly interconnected genetic network (*n*=65 loci; *q*<0.05) associated with greater levels of substance use during adolescence, as well as an earlier age of onset amongst users. Associations were specific to the neonatal period and not observed at age 7. Key annotated genes included *PACSIN1, NEUROD4* and *NTRK2*, implicated in neurodevelopmental processes. Several of the identified loci were associated with known methylation quantitative trait loci, and consequently likely to be under significant genetic control. Collectively, these 65 loci were also found to partially mediate the effect of prenatal maternal tobacco smoking on adolescent substance use. Together, findings lend novel insights into epigenetic correlates of substance use, highlight birth as a potentially sensitive window of biological vulnerability and provide preliminary evidence of an indirect epigenetic pathway linking prenatal tobacco exposure and adolescent substance use.

## Introduction

Substance abuse is a major public health concern that incurs heavy costs to individuals, their families and wider society. Collectively, it is estimated that one in ten of all fatalities result from harmful use of alcohol, tobacco and illicit drugs, representing one of the leading preventable causes of death worldwide.^1^ In addition, substance abuse is associated with a range of negative outcomes that compromise quality of life and long-term productivity, including psychiatric illness, disability, criminality and unemployment.^2, 3^ Consequently, a key challenge for research is to identify factors that drive individual susceptibility to substance abuse, to inform effective prevention and early intervention strategies.^4^

Like most complex phenotypes, substance abuse results from a dynamic interplay of genetic and environmental influences. Studies have shown that the heritability of substance-use disorders is moderate to high (~49–70%)^5^ and that this genetic vulnerability interacts with environmental risk exposure. Indeed, epidemiological studies have identified a number of pre- and postnatal factors associated with substance abuse risk, including substance exposure during pregnancy, parental psychopathology and criminality, low socioeconomic status, childhood maltreatment and affiliation with delinquent peers.^6^ However, the biological mechanisms through which these effects are mediated are poorly understood.

In recent years, epigenetic processes that regulate gene expression^7^ have emerged as a potential mechanism of interest. One of these processes, DNA methylation (DNAm), has received increasing attention. DNAm modulates transcription via to the addition of a methyl group to DNA base pairs, primarily in the context of cytosine–guanine (CpG) dinucleotides.^7^ Studies have shown that (i) DNAm is affected by genetic variability, as demonstrated by the discovery of a large number of methylation quantitative trait loci (mQTLs);^8, 9^ (ii) DNAm is also sensitive to pre- and postnatal environmental influences, including nutritional, chemical and psychosocial factors (for example, prenatal tobacco exposure);^10, 11^ and (iii) aberrant patterns of DNAm have been linked to a wide range of physical and psychiatric disorders, including addiction.^12^ For example, animal studies have shown that repeated drug administration (for example, alcohol and cocaine) can lead to DNAm changes in reward-related regions of the brain (for example, striatum).^13^ In turn, these changes can influence the expression of genes involved in synaptic plasticity and memory consolidation, driving neuroadaptations that underlie the onset and persistence of addictive behaviors.^14^ Importantly, drug-induced epigenetic changes have been found to occur as early as gestation.^12^ For example, a recent study in mice reported an epigenetically-mediated effect of early nicotine exposure on pup's neural structure and behavior, which persisted into adulthood.^15^ So far, studies in humans have provided initial support for animal findings, reporting methylomic differences between substance abusers and drug-free controls across several tissue types and substances.^12, 16^

Despite these promising findings, current research in humans has been limited in four key ways.^17^ First, the vast majority of studies have examined adult samples already exposed to substances. As a result, it has not been possible to establish whether altered DNAm patterns are a risk factor for and/or consequence of substance use. Disentangling these associations is essential to better delineate the role of epigenetic mechanisms in addiction risk and to enable the identification of novel therapeutic targets. Second, because existing studies have typically included DNAm data at a single time point, it is unclear whether epigenetic effects may be observable across time or only during specific developmental periods. This is particularly relevant given that DNAm has been shown to be highly dynamic across the lifespan, enabling cells to respond to changing internal and external inputs.^18^ As such, clarifying how DNAm associates with substance use over time may provide important insights into windows of biological vulnerability. Third, little is known about what genetic and environmental factors may underlie variability in DNAm patterns associated with substance use. Characterizing these potential influences may not only offer valuable opportunities for preventative intervention, but also make it possible to test the role of the epigenome as a potential mediator in the link between risk exposure and later substance use. Finally, existing studies have primarily focused on one type of substance at a time. Although substance-specific risk factors have been identified, evidence from both genetically-informative and epidemiological studies indicate that substance-use risk across drug classes is largely accounted for by a common underlying liability dimension.^5, 19^ Consequently, examining epigenetic markers common to multiple substances, in addition to substance-specific markers, may help shed further light into the biological basis of substance-use liability.

To address these gaps in the literature, we believe we conducted the first genome-wide, prospective study to examine associations between DNAm in early life (that is, collected at repeated time points pre-substance-use initiation; birth and age 7) and substance use in adolescence (measured as a latent factor spanning tobacco, cannabis and alcohol use). Our aim was to address the following key questions:

1. Are DNAm patterns at birth prospectively associated with adolescent substance use?
2. Are these associations stable across early childhood (birth to age 7)?
3. Do the identified DNAm markers associate with genetic and environmental influences?

## Materials and methods

### Participants

The Epigenetic Pathways to Conduct Problems Study consists of a subsample of youth (*n*=339) drawn from the Avon Longitudinal Study of Parents and Children (ALSPAC) who (i) have repeated measures of DNAm and (ii) follow previously established trajectories of conduct problems (4–13 years).^20^ Only youth who had complete substance-use ratings (age 14–18) as well as epigenetic data at birth and age 7 (*n*=244, 54% female) were included in the present study. ALSPAC is an ongoing epidemiological study of children born between 1991–92 from 14 541 women residing in Avon, UK. Of these initial pregnancies, there was a total of 14 676 fetuses, resulting in 14 062 live births and 13 988 children who were alive at 1 year of age.^21^ When compared with 1991 National Census Data, the ALSPAC sample was found to be broadly similar to the UK population as a whole.^22^ Informed consent was obtained from all ALSPAC participants and ethical approval was obtained from the ALSPAC Law and Ethics Committee and the Local Research Ethics Committees. Please note that the study website contains details of all the data that is available through a fully searchable data dictionary: http://www.bris.ac.uk/alspac/researchers/data-access/data-dictionary/.

### Measures

#### Adolescent substance use

Substance use was assessed via self-report ratings of tobacco and cannabis use (age 14, 16 and 18 using frequency items ranging from ‘never' to ‘daily'), as well as alcohol use (age 16 and 18 using the 10-item alcohol use disorders identification test).^23^ Confirmatory factor analysis was used to extract (i) three first-order factors of tobacco, cannabis and alcohol use, accounting for shared variance across time points for each of these substances; and (ii) a single second-order factor of substance use, accounting for shared variance between substances *and* across time. The factor model showed adequate fit: *χ*^2^ (ref. 18)=49.55; *P*<0.01; comparative fit index=0.91; Tucker–Lewis index=0.86; root mean square error of approximation=0.08, 90% confidence intervals (CIs)=0.05, 0.10; with standardized loadings ranging from 0.58 to 0.96 (Supplementary Figure 1).

#### DNA methylation data

A total of 500 ng high molecular weight genomic DNA from blood (cord at birth, whole at age 7) was bisulfite-converted using the EZ-DNA methylation kit (Zymo Research, Orange, CA, USA). DNAm was quantified using the Illumina HumanMethylation450 BeadChip (Illumina, San Diego, CA, USA) with arrays scanned using an Illumina iScan (software version 3.3.28). Initial data quality control was conducted using GenomeStudio (San Diego, CA, USA; version 2011.1) to determine the status of staining, hybridization, target removal, bisulfite conversion, specificity, non-polymorphic and negative controls. Samples that survived this stage were quantile normalized using the dasen function within the wateRmelon 1.0.3 package^24^ in R and batch-corrected using the ComBat package.^25^ Probes were removed if they were cross-reactive, used for sample identification on the array, or had a single-nucleotide polymorphism at the single-base extension with a minor allele frequency larger than 5% (that is, common polymorphisms), leaving a total of 413 510 probes.^26, 27^ DNAm levels are indexed by beta values (ratio of: methylated signal/ methylated+unmethylated signal).

#### Prenatal environmental risks

We included prenatal risks that have been previously linked to adolescent substance use, including maternal prenatal smoking, alcohol use and exposure to stressful events.^6^ Maternal smoking and alcohol use during the first trimester of pregnancy were measured via maternal ratings, using a yes/no binary variable for smoking (for biological validation see the results section), and a 4-point scale for alcohol use (‘never' to ‘daily'). With regards to stress exposure, we included cumulative risk scores of prenatal (18–32 weeks) adversity covering the following four domains: (i) life events (for example, death in family and accident); (ii) contextual risks (for example, poor housing and financial problems); (iii) parental risks (for example, psychopathology and criminal behavior); and (iv) interpersonal risks (for example, partner abuse and family conflict). These cumulative risk scores were estimated using confirmatory factor analysis based on maternal reports, as described elsewhere.^28^

### Data analysis

Analyses were performed in R (version 3.0.1)^29^ and Mplus (version 6.1.1)^30^ adjusting for sex and cell-type proportions (CD8 T-lymphocytes, CD4 T-lymphocytes, natural killer cells, B-lymphocytes, monocytes), estimated using the reference-based approach detailed in Houseman *et al.*^31^

#### Step 1: Are DNAm patterns at birth associated with adolescent substance use?

Genome-wide association analyses between DNAm at birth and substance use were performed using the IMA package.^32^ Differentially methylated probes (DMPs) passing a false discovery rate (FDR) correction of *q*<0.05 were considered significant. These DMPs were then uploaded to the UCSC genome browser (GRCh37/hg19 assembly)^33^ to explore their potential functional relevance, by comparing their genomic location to that of key regulatory elements recorded in the Encyclopedia of DNA Elements (ENCODE) database (http://genome.ucsc.edu/ENCODE/), including (i) transcription factor binding sites (data generated on 161 transcription factors in 91 cell types via ChIP-seq); (ii) DNase I hypersensitivity clusters (based on data from 125 cell types) and (iii) histone marks (only relevant cell lines examined, including blood [GM12878, K562] and umbilical vein endothelial [HUVEC] cells).

Genes to which DMPs were annotated were then examined to identify (i) underlying genetic networks, using the GeneMANIA bioinformatics software, which is based on known genetic and physical interactions, shared protein domains as well as co-expression data (http://www.genemania.org; see Supplementary Table 1); and (ii) enriched biological pathways, by using an optimized gene ontology method that controls for a range of potential confounds, including background probe distribution and gene size (Supplementary Table 1).

As a supplement to the probe-level analysis, we also used the Comb-p application within Python^34^ with default settings (*P* threshold: 1.00E−04; sliding window size: 500 bp), to identify wider differentially methylated regions based on spatially-correlated *P*-values.

#### Step 2: Are these associations stable across early childhood (that is, birth to age 7)?

Given that DNAm is temporally dynamic^18^—particularly in early development^9^—markers identified at one time point may not necessary continue to be associated with substance use at other time points. To test this, we examined whether DMPs identified in step 1 (that is, birth) were also significantly associated of adolescent substance use at age 7 (that is, follow-forward approach; FDR-corrected *q*<0.05).

#### Step 3: Do these markers relate to genetic and environmental influences?

As a last step, we investigated potential genetic and environmental factors that may influence DNAm levels of the identified DMPs. Given that our sample was underpowered to directly examine genetic polymorphisms (that is, single-nucleotide polymorphisms) affecting DNAm, we used the ALSPAC-derived mQTLdb resource (http://www.mqtldb.org/) to search for known mQTLs associated with our DMPs (see Supplementary Table 1 for further details). Potential environmental influences were examined next by testing associations between prenatal exposures and DMPs. Because of the large number of DMPs identified, we grouped these into a single, cumulative DNAm risk score to minimize multiple testing burden. Specifically, we applied a method typically used for polygenic risk scores,^35^ where we multiplied the methylation values of our DMPs by their respective standardized regression betas (that is, weights), and then summed these together into a DNAm risk score. This approach enabled us to reduce the volume of our methylation data, whereas the use of weights ensured that DMPs maintained their relative predictive importance (as opposed to alternative approaches, for example, averaging DNAm levels across DMPs). Once calculated, we examined associations between this DNAm risk score and prenatal exposures, using Pearson's bivariate correlations. Significant prenatal risks (*q*<0.05) were then incorporated into a single path analytic model in Mplus (maximum likelihood estimation), together with the DNAm risk score and the substance use factor, to test for indirect effects. Associations in the model were considered significant if they survived bootstrapped CIs (10 000 times).^36^ Significant paths (prenatal risks → DNAm → substance use) were tested for an indirect effect using bootstrapped model constraint statements.

#### Code availability

Computer code used in our analyses is available from the authors on request.

## Results

### Epigenome-wide association analysis at birth

**Figure 1:**
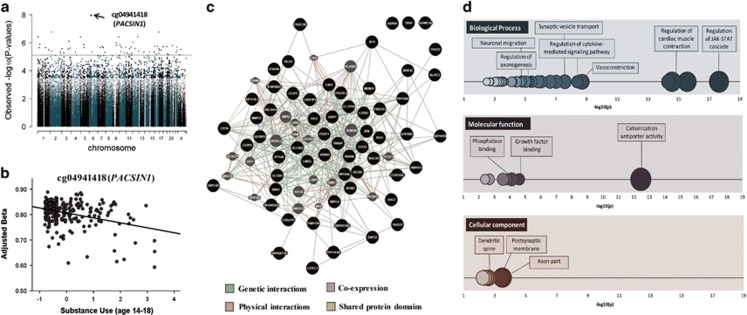
Differentially methylated loci at birth associated with adolescent substance use. (**a**) Manhattan plot showing genome-wide associations between DNA methylation at birth and later substance use (age 14–18). The dotted line represents the false discovery rate (FDR)-correction threshold (i.e., loci above the line are considered significant). (**b**) Prospective association between the top differentially methylated locus at birth and later substance use. The *X* axis shows substance use factor scores, whereas the *Y* axis represents beta methylation values, adjusted for sex and cell-type proportions. (**c**) Gene network analysis using GeneMANIA. Black circles represent genes (*n*=60) associated with the 65 probes found to be related to adolescent substance use in the genome-wide analysis at birth. Gray circles represent additional genes predicted by GeneMANIA based on genetic and physical interactions, shared protein domains as well as protein co-expression data. The gene network analysis demonstrates that, rather than being isolated, these genes clustered into a complex interconnected network. (**d**) Significantly enriched biological processes (blue), molecular functions (purple) and cellular components (red), based on gene ontology (GO) analysis of 60 genes annotated to probes that predict substance use at birth (*n*=65; *q*<0.05). Circles represent GO terms that survive FDR correction and contain at least one gene. The *X* axis represents −log(10) *P*-values. The opacity of the circles indicates level of significance (darker=more significant). The size of the circles indicates the percentage of genes in our results for a given pathway compared with the total number of genes in the same pathway (i.e., larger size=larger %).

At birth, 65 probes prospectively associated with adolescent substance use after genome-wide correction (*q*<0.05; Table 1 and Figure 1a). Of these DMPs, 33 were ‘hypomethylated' (that is, lower DNAm associating with higher substance use), whereas the other 32 were ‘hypermethylated' (that is, higher DNAm associating with higher substance use). Overall, DMPs were most frequently located in the gene body (40%) or promoter region near the transcription start site (30% see Supplementary Table 2). DNAm levels were significantly interrelated across the majority of DMPs (76% of correlations=*q*<0.05; *r*_max_=0.58; *r*_min_=−0.52; *r*_absolute average_=0.20; see Supplementary Table 3). The most significant probe, cg04941418 (*P*=1.10E−08; *q*=0.005, Figure 1b), is located in *PACSIN1*, a developmentally regulated gene that has an important role in synaptic neurotransmission, axonal growth and dendritic branching.^37, 38^ Other annotated genes in the table include (i) *SHC2* (cg02290110) and *NTRK2* (cg01009697), both implicated in neuronal neurotrophin-activated Trk receptor signaling,^39, 40^ (ii) *CLSTN1* (cg07395930), involved in calcium-mediated post-synaptic signals, and (iii) *NEUROD4* (cg20056324), involved in neural differentiation. DMPs were then uploaded in Genome Browser for functional characterization, based on ENCODE data on regulatory elements. All DMPs overlapped with histone marks; 62% (*n*=40) coincided with transcription factor binding sites; and 57% (*n*=37) were located within DNAse I hypersensitive clusters. Overall, 48% (*n*=31) of DMPs were mapped to all three regulatory elements (Supplementary Table 4).

DMPs were annotated to a total of 60 genes, which were examined further to identify underlying genetic networks and enriched biological pathways. On the basis of GeneMANIA analysis, 57 of the 60 genes were connected to form a compact cluster network (Figure 1c). Our gene ontology analysis also indicated that these genes are involved in a range of biological processes, including regulation of JAK-STAT cascade, vasoconstriction, cytokine-mediated signaling and axonogenesis (2.30E−18*<P*<3.37E−03; Figure 1d). Of note, enriched cellular components included axon part, post-synaptic membrane and dendritic spine (2.94E−04*<P*<3.09E−03; for the full list of GO terms, see Supplementary Table 5).

Results from the Comb-p analysis indicated that there were no significant differentially methylated regions after genome-wide correction.

### Follow-forward at age 7

None of the DMPs identified at birth continued to prospectively associate with adolescent substance use by age 7, after multiple correction (*q*>0.05; Supplementary Table 6). Two DMPs showed nominal associations (cg02404636 [*SFI1*]: *Std B*=0.21, *P=*0.001; cg20056324 [*NEUROD4*]: *Std B*=0.13, *P=*0.05), both following the same direction of effects observed at birth. Given this lack of temporal stability, we proceeded to test, for each DMP, how much DNAm levels at birth correlated with those at age 7 (that is, autocorrelation). We found that only 12 DMPs (18%) showed an autocorrelation significant at *P*<0.05, 11 of which were in the positive direction (across all DMPs: *r*_max_=0.67; *r*_min_=−0.13; *r*_absolute average_=0.07). Interestingly, however, the pattern of intercorrelations across DMPs at age 7 resembled that observed at birth (Supplementary Figure 2). In other words, whereas DMPs typically did not correlate with themselves over time, the way in which they correlated with each-other within time points was very similar, potentially reflecting a similar underlying co-methylation network.

### Genetic and environmental influences

**Figure 2:**
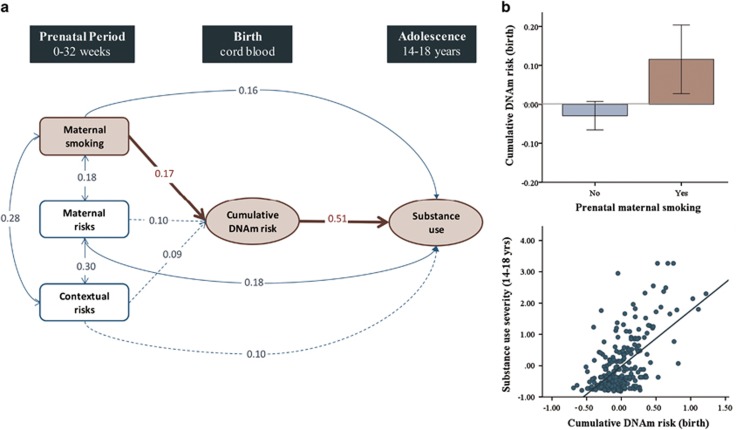
Indirect effect of prenatal smoking on adolescent substance use via neonatal DNA methylation. (**a**) Path analytic indirect effects model. Dotted arrowed lines indicate non-significant paths. Single arrowed lines indicate standardized path coefficients that survived bootstrap-corrected confidence intervals (i.e., significant path). Red arrows show significant indirect path. Population effect sizes are interpreted using the standardized estimates (Std. B) following Cohen's guidelines: an effect of 0.10 is small effect, an effect of 0.24 is a medium effect, and an effect of 0.37 is a large effect. (**b**) Graphical representation of the indirect effect, where prenatal smoking associates with higher cumulative DNA methylation risk at birth (top panel), which in turn associates with higher substance use in adolescence (bottom panel). DNAm, DNA methylation.

The 65 DMPs identified at birth were carried forward to explore associations with potential genetic and environmental influences. On the basis of the mQTLdb search, we found that five of the DMPs were associated with known mQTLs (*n*_cis_=4; *n*_trans_=1), suggesting that DNAm levels across these sites are likely to be under considerable genetic control (Supplementary Table 4). Of note, temporal stability of these DMPs was stronger (*r*_average_=0.25) than the average across all DMPs noted above, consistent with what has previously been observed at the genome-wide level.^9^ With regards to environmental influences, we found that three prenatal exposures significantly correlated with DNAm (measured as a cumulative risk score comprising of all DMPs)—maternal tobacco smoking, maternal risks and contextual risks (Table 2). To test for indirect effects, we estimated a path analytic model (Figure 2a) that included these three prenatal exposures, the cumulative DNAm risk score, and the adolescent substance use outcome. Maternal smoking was the only prenatal factor to uniquely associate with higher cumulative DNAm risk (over and above other exposures), which in turn associated with higher substance use in adolescence (Figure 2b). Analysis of this pathway indicated a significant indirect effect of maternal smoking on substance use, via cumulative DNAm risk (unstandardized *b*=0.19, s.e.=0.07, *P*=0.01, bootstrapped 95% CI=0.05–0.37). To minimize the possibility that associations with prenatal exposures may simply reflect genetic confounding, we reran analyses using a cumulative DNAm risk score that did not include any of the DMPs associated with mQTLs (that is, five probes removed). This score was highly correlated with the original score (*r*=0.99; *P*=3.88E−252) and findings remained consistent.

### Follow-up analyses

#### *PACSIN1*: relevance to the brain

*PACSIN1*_cg04941418_ emerged as the top DMP at birth to associate with adolescent substance use. Given that our DNAm data was extracted from peripheral blood, we used the Genotype-Tissue Expression project portal (GTEx; http://www.gtexportal.org/home/; ^41^ and the EMBL-EBI Expression Atlas (https://www.ebi.ac.uk/gxa/home/);^42^ to assess *PACSIN1* expression across tissues. *PACSIN1* was found to be most highly expressed in brain tissue, including regions implicated in drug-seeking behavior and addiction risk, such as the prefrontal cortex, nucleus accumbens, amygdala and hippocampus (Supplementary Figure 3). We then used the BrainCloud tool (http://braincloud.jhmi.edu/plots/),^43^ to trace the developmental course of *PACSIN1* expression across the lifespan (fetal–age 80), based on postmortem prefrontal cortex tissue from 269 healthy subjects. The resulting plot showed that the most dramatic change in expression levels occurs during the neonatal period, bridging lower expression levels during fetal development with a higher, stable trajectory of expression from around 3 months of age onward (Supplementary Figure 3).

#### Age of substance-use onset

Overall, 65 DMPs at birth prospectively associated with substance-use severity in adolescence. As a sensitivity analysis, we additionally tested whether these DMPs also associated with age of onset among substance users. On the basis of three items that combined self-report data across age 16 and 18, we found that, within youth who endorsed using substances, higher cumulative DNAm risk correlated with lower reported age when first ‘smoked whole cigarette' (*r*=−0.19, *P*=0.03, *n*_endorse_=129), ‘tried cannabis' (*r*=−0.36, *P*=3.40E-04, *n*_endorse_=93) and ‘had whole alcoholic drink' (*r*=−0.23, *P*=0.001, *n*_endorse_=195), respectively. For data on frequencies, correlations and details about how the items were created, see Supplementary Table 7.

#### Indirect effects for specific substances

We found a significant indirect effect of prenatal tobacco smoking on adolescent substance use, via cumulative DNAm risk. Here, we wanted to clarify whether this indirect effect was observed across all substances or only specific ones (for example, adolescent tobacco use). To this end, we reran the path analysis with the three first-order factors of tobacco, cannabis and alcohol use (Supplementary Figure 4). Indirect effects were significant across all three substance types (tobacco: *b*=0.31, s.e.=0.12, *P*=0.01, bootstrapped 95% CI=0.09–0.59; cannabis: *b*=0.71, s.e.=0.29, *P*= 0.01, bootstrapped 95% CI=0.22–1.34; alcohol: *b*=0.14, s.e.=0.06, *P*=0.03, bootstrapped 95% CI=0.04–0.30). Because the first-order factor of cannabis use contained one outlier (that is, >3 s.d. from the mean), the analysis was also rerun with winsorized data for this score and results remained consistent.

#### Biological validation of prenatal maternal smoking

Finally, to ensure the validity of our measure of prenatal smoking—which was derived from a single yes (*n*=48) /no (*n*=213) item reported by mothers—we ran an epigenome-wide analysis with prenatal smoking predicting neonatal DNAm. As expected, the top differentially methylated locus was cg05575921 (*AHRR; P*=6.96e−16; *q*=2.88E−10, see Supplementary Table 8), a well-established, sensitive and specific biomarker of tobacco exposure.^10, 11, 44^ Of note, there was no overlap between the maternal smoking and adolescent substance-use DMPs.

## Discussion

The aim of this study was to characterize DNA methylation patterns prospectively associated with substance-use risk, using longitudinal data spanning gestation to adolescence. We highlight here three key findings: (i) epigenetic variation across 65 loci at birth associated with higher tobacco, cannabis and alcohol use in adolescence, as well as an earlier age of substance-use onset; (ii) these effects were specific to the neonatal period and not observed in mid-childhood; and (iii) several of the identified loci were associated with known genetic mQTLs, and all, collectively, mediated the effect of prenatal tobacco smoking on adolescent substance use. These findings lend novel insights into epigenetic predictors of substance use, highlight birth as a potentially sensitive window of biological vulnerability and provide preliminary support for the role of DNAm as an indirect pathway linking prenatal exposures to adolescent behavioral outcomes.

### Epigenetic variation at birth associates with substance use in adolescence

Although the impact of substance use on DNAm has been repeatedly documented,^12^ less is known about the extent to which DNAm may confer risk for substance use, as existing studies have typically focused on adults already exposed to substances. To our knowledge, this is the first study to address this gap by examining DNA collected before substance-use initiation. Furthermore, the use of a latent factor score comprising of tobacco, cannabis and alcohol use enabled us to examine the potential role of methylomic variation in broader substance-use liability. On the basis of genome-wide analyses, we found that epigenetic variation across 65 loci at birth associated with higher substance use 14–18 years later, as well as an earlier age of onset among substance users. These loci were annotated to genes that, together, formed a compact underlying genetic network and were enriched for a range of biological pathways, including neural processes (for example, axonogenesis and synaptic transport) and cellular components (for example, axon, dendritic spine and post-synaptic membrane). The most differentially methylated locus was annotated to *PACSIN1*, a developmentally-modulated gene that has an important role glutamate neurotransmission, axonal growth, dendritic branching and synaptic plasticity^45^ and that is highly expressed in brain tissue,^37, 38^ including regions implicated in drug-seeking behavior and addiction risk (for example, nucleus accumbens, frontal cortex, amygdala and hippocampus).^46^ Other key annotated genes also implicated in early brain development included *NEUROD4*, involved in neuronal differentiation, and *NTRK2*, a Trk receptor for multiple neurodevelopmental genes, including brain-derived neurotrophic factor, neutrophin 4 and nerve growth factor.^40^

### The neonatal period as a potential window of biological vulnerability

The inclusion of repeated DNAm measures enabled us to test the stability of epigenetic effects during childhood. We found that none of the loci identified at birth continued to predict substance use by age 7 (after multiple correction). This specificity of effects around birth is consistent with previous studies from our group examining longitudinal associations between DNAm and developmental outcomes.^28, 47, 48^ Findings are also consistent with a recent study based on the ALSPAC sample that reported low genome-wide continuity in DNAm patterns over time,^9^ especially when comparing birth to other time points. A number of factors may drive the temporal differences observed. First, findings may reflect tissue-specific DNAm patterns, as data was extracted from two different blood sources (cord blood at birth vs whole blood at age 7). Second, differences may reflect the specific timing of environmental influences, whereby methylation patterns at birth may be a more reliable proxy for intra-uterine risk exposures and associated perturbations in fetal development,^49^ compared with age 7. Third, the neonatal period may represent a particularly sensitive window of biological vulnerability to future substance use. For example, epigenetic patterns at birth may trigger downstream developmental consequences resulting in enduring individual differences (for example, in neural networks underlying drug-seeking behavior and addiction)^12^^,^^15^ without the epigenetic signature being maintained over time.^18^ Given that we still know little about the role of tissue differences, environmental influences and developmental processes on DNAm,^50^ the above explanations are inevitably speculative and will necessitate further investigation.

### Genetic and environmental influences on DNAm patterns associated with substance use

The identification of neonatal DNAm patterns associated with adolescent substance use raises questions about what kind of factors may drive this methylomic variation in the first place. Evidence suggests that DNAm patterns^8, 10^—like substance use liability^5, 6^—reflect the influence of both genetic and environmental factors. On the basis of data from mQTLdb,^9^ we found that 5 of our 65 DMPs were associated with known mQTLs, suggesting that they may be considerably influenced by genetic structure. Although these associations point to potentially large genetic effects on a relatively small number of our DMPs, it is important to note that the heritability of DNAm patterns is greater than what can currently be explained using known mQTLs.^9^ As such, genetic effects on our other DMPs cannot be ruled out, especially the presence of polygenic effects. With regards to environmental influences, we found that three prenatal factors were associated with cumulative DNAm risk at birth (comprising all DMPs): maternal tobacco smoking (measured in the first trimester), maternal risks (for example, psychopathology and criminal behavior) and contextual risks (for example, poor housing and financial problems). Associations remained consistent after removing any mQTL-related DMP from our DNAm risk score to minimize genetic confounding. These findings support the presence of both genetic and environmental influences on substance use related DNAm patterns. It is important to note, however, that because associations were based on correlational analyses, they should be interpreted with caution and considered more as well-grounded hypotheses for further examination in larger longitudinal studies.

### DNAm as an indirect pathway linking prenatal smoking to adolescent substance use

We found that one prenatal exposure—maternal tobacco smoking—uniquely associated with substance use over and above other exposures, and that this association was partially mediated by cumulative DNAm risk at birth. Importantly, this indirect effect was observed across all three substance types (not just tobacco use, but also cannabis and alcohol use)—pointing to a potential link between prenatal tobacco exposure and broader substance-use liability. To our knowledge, this is the first example in humans of an indirect effect of prenatal exposures on substance-related outcomes via DNAm, consistent with recent work reported in animals.^15^ However, due to the correlational nature of the analyses, such evidence should be considered preliminary and in need of rigorous assessment using advanced causal inference methods (for example, two-step Mendelian randomization).^51, 52^ In particular, further work will be needed to trace the specific biological pathways through which this indirect effect may be expressed. Experimental studies have shown that prenatal nicotine exposure causes neuromorphological changes (for example, dendritic branching, axonal growth and spine density) in brain circuits underlying motivation, learning and reward-processing, which in turn confer latent vulnerability for substance use and other externalizing problems (for example, hyperactivity and aggression).^15, 53, 54^ As such, it will be of interest to test whether the observed effect of prenatal nicotine exposure on substance use may be expressed via epigenetically-modulated changes in neural development, organization and structure. This will also require a more comprehensive investigation of DNAm in the context of other epigenetic processes, which have also been implicated in substance use and addiction (for example, histone modifications and microRNAs, see Nestler^14^ for a review).

### Limitations and future directions

Findings should be interpreted in light of a number of limitations. First, the current study was based on a modestly sized population-based sample of youth. At present, ALSPAC is the only cohort, to our knowledge, that is prospective enough to enable the examination of neonatal DNAm patterns associated with adolescent substance use. Consequently, we were unable to replicate our results in an independent sample. In future, it will be important to test the robustness of findings using other epidemiological cohorts, as well as establishing the relevance of the identified markers in the development of more severe clinical phenotypes, including substance abuse and dependence. Second, findings were based on DNAm from peripheral samples; as such, more research will be needed to establish the relevance of the identified markers to brain function. Future studies incorporating imaging data will be important for establishing whether these markers associate with structural or functional alterations in addiction-relevant neural pathways (for example, related to reward-processing, impulse control, learning and memory), contributing to a more mechanistic understanding of the identified associations. Third, functional characterization of the DMPs was performed using ENCODE data, as we did not have access to RNA. Integration of transcriptomic data will mark an important step toward establishing the downstream effects of the observed DNAm changes on gene expression. Fourth, despite the fact that we identified prospective associations between DNAm and substance use (that is, DNAm collected before initiation of substance use), it is not possible to establish causality. Finally, the study focused exclusively on DNA methylation, and other epigenetic processes (for example, histone modifications and microRNAs) are likely to be important in mediating the onset and consequences of addiction.^14^

## Conclusions

The present findings lend novel insights into early epigenetic correlates of substance use, pinpointing specific markers for future interrogation. Evidence of temporally-specific effects points to birth as a potentially sensitive window of biological vulnerability, which may particularly benefit from intervention efforts. Findings also highlight prenatal smoking as an important prevention target, and contribute to a better understanding of the biological mechanisms through which tobacco exposure during pregnancy may increase risk for future substance use.

## Figures and Tables

**Table 1:** DNA methylation loci at birth that prospectively associate with substance use in adolescence (*n*=65, *q*<0.05)

*Probe*	*Gene*	*Chr*	*Genomic location*	*Position*	*Std B*	P*-value*	*FDR (*q*-value)*
cg04941418	*PACSIN1*	6	5′UTR	34493129	−0.34	1.10E−08	0.005
cg11074746	*MEMO1*	2	TSS1500	32236343	0.31	1.77E−07	0.02
cg00335219	*—*	16	—	86012305	0.27	1.79E−07	0.02
cg07395930	*CLSTN1*	1	Body	9791419	−0.31	3.84E−07	0.02
cg01589998	*FOXN4*	12	Body	109729478	0.31	3.92E−07	0.02
cg13978601	*PPP2R4*	9	Body	131905041	−0.31	4.10E−07	0.02
cg23361356	*SLC9A3*	5	Body	508834	−0.31	4.17E−07	0.02
cg27020216	*SGEF*	3	Body	153840347	0.29	8.35E−07	0.03
cg14632140	*LMO3*	12	5′UTR	16758112	0.29	9.82E−07	0.03
cg08080985	*C9orf95*	9	Body	77703079	0.30	1.07E−06	0.03
cg20685020	*ATP6V0B*	1	5′UTR	44440676	0.30	1.34E−06	0.03
cg25229198	*ADAMTS6*	5	Body	64660684	−0.30	1.38E−06	0.03
cg05968179	*USP6NL*	10	Body	11505706	−0.29	1.42E−06	0.03
cg01009697	*NTRK2*	9	TSS1500	87283470	0.29	1.56E−06	0.03
cg19026817	*—*	20	—	56782259	−0.29	1.64E−06	0.03
cg05116255	*ANKRD30A*	10	TSS1500	37413782	−0.29	1.85E−06	0.03
cg04799664	*NLRC5*	16	5′UTR	57053850	−0.29	1.85E−06	0.03
cg07746699	*IFT140*	16	TSS200	1662305	0.30	1.87E−06	0.03
cg02290110	*SHC2*	19	TSS1500	461808	0.29	1.91E−06	0.03
cg06951646	*TRRAP*	7	Body	98586548	−0.29	1.92E−06	0.03
cg02052845	*HGS*	17	Body	79658835	−0.29	2.00E−06	0.03
cg05033322	*ATM*	11	TSS1500	108093245	0.29	2.08E−06	0.03
cg02404636	*SFI1*	22	TSS1500	31891804	0.29	2.10E−06	0.03
cg05620865	*AP3B1*	5	Body	77588408	−0.29	2.22E−06	0.03
cg14291256	*MMP21*	10	Body	127461065	−0.29	2.25E−06	0.03
cg12593849	*STX12*	1	3′UTR	28150710	−0.29	2.31E−06	0.03
cg10844884	*ZNF22*	10	Body	45498904	−0.29	2.32E−06	0.03
cg27242945	*CAV1*	7	1st exon	116165134	0.28	2.35E−06	0.03
cg13562386	*TAGLN2*	1	TSS1500	159895724	0.29	2.36E−06	0.03
cg25767859	*PKD1L1*	7	Body	47859324	−0.29	2.37E−06	0.03
cg12202228	*FNDC3B*	3	Body	171871829	−0.29	2.52E−06	0.03
cg20056324	*NEUROD4*	12	TSS200	55413610	0.29	2.60E−06	0.03
cg27122536	*FOXF1*	16	1st exon	86544658	0.29	2.67E−06	0.03
cg14661886	*PRRT3*	3	TSS200	9994197	0.29	2.75E−06	0.03
cg27596068	*SERPINH1*	11	TSS1500	75272301	−0.28	2.77E−06	0.03
cg23088142	*—*	3	—	137531265	0.29	2.99E−06	0.03
cg08625693	*DLG3*	X	Body	69674126	−0.26	3.09E−06	0.03
cg05463325	*RAB4A*	1	TSS1500	229406534	0.29	3.23E−06	0.04
cg01894322	*PPP1R1B*	17	Body	37789575	−0.28	3.38E−06	0.04
cg13138952	*RPH3AL*	17	Body	236013	−0.29	3.39E−06	0.04
cg01338630	*RASD2*	22	3′UTR	35948166	−0.27	3.77E−06	0.04
cg22409100	*SLC8A1*	2	TSS1500	40658918	0.14	4.01E−06	0.04
cg24954684	*CLIP1*	12	TSS1500	122907641	0.27	4.08E−06	0.04
cg22467567	*IGFBP5*	2	5′UTR	217559885	0.28	4.39E−06	0.04
cg25179876	*NRP1*	10	Body	33483109	−0.19	4.47E−06	0.04
cg04388666	*CCDC85C*	14	TSS1500	100072073	0.28	4.61E−06	0.04
cg13244417	*TMTC4*	13	TSS200	101327186	0.28	4.75E−06	0.04
cg16661000	*HAPLN3*	15	5′UTR	89437710	0.27	4.81E−06	0.04
cg00276455	*—*	6	—	54904638	−0.28	4.82E−06	0.04
cg08261702	*LOC728743*	7	Body	150103112	0.25	5.02E−06	0.04
cg05168344	*LZTR1*	22	Body	21340160	−0.28	5.07E−06	0.04
cg02725795	*FAM175A*	4	Body	84405871	0.27	5.18E−06	0.04
cg20336014	*MGEA5*	10	TSS200	103578255	0.27	5.49E−06	0.04
cg26047334	*TNS1*	2	5′UTR	218785909	0.25	5.56E−06	0.04
cg18769584	*LZTFL1*	3	3′UTR	45866619	−0.27	5.64E−06	0.04
cg13083436	*LUZP2*	11	TSS200	24518414	0.28	5.74E−06	0.04
cg23530543	*HEPN1*	11	TSS1500	124788414	−0.28	5.98E−06	0.04
cg02957771	*FBXO31*	16	Body	87380349	−0.27	6.13E−06	0.04
cg17396676	*EPS15L1*	19	TSS1500	16583990	−0.28	6.56E−06	0.04
cg09278687	*TBX6*	16	Body	30100430	−0.28	6.91E−06	0.04
cg20643362	*C19orf12*	19	1st exon	30206369	0.27	7.37E−06	0.04
cg10395806	*CTTN*	11	Body	70280601	−0.28	7.43E−06	0.04
cg00877150	*BCOR*	X	5′UTR	39972039	−0.27	7.52E−06	0.04
cg20319698	*LRRFIP1*	2	Body	238644099	−0.27	7.73E−06	0.04
cg14712611	*ANK2*	4	5′UTR	113739379	0.27	7.74E−06	0.04

Abbreviation: FDR, false discovery rate.

**Table 2:** Associations between prenatal exposures, cumulative DNAm risk at birth and adolescent substance use

	*Prenatal exposures*											
	*Maternal smoking*		*Maternal alcohol use*		*Maternal risks*		*Family risks*		*Contextual risks*		*Life events*	
	r	P	r	P	r	P	r	P	r	P	r	P
Cumulative DNAm risk (birth)	0.20	1.21E−03	−0.07	0.28	0.15	0.01	−0.03	0.59	0.16	0.01	−0.07	0.26
Substance use (age 14–18)	0.32	1.58E−07	−0.03	0.60	0.32	1.44E−07	0.09	0.15	0.28	4.33E−06	0.03	0.61

Abbreviation: DNAm, DNA methylation.

## References

[bib1] UNODC. World drug report. In: UNOoDa (ed). Crime. United Nations: New York, NY, USA, 2013 p 87.

[bib2] Rehm J, Mathers C, Popova S, Thavorncharoensap M, Teerawattananon Y, Patra J.. Global burden of disease and injury and economic cost attributable to alcohol use and alcohol-use disorders. Lancet 373: 2223–2233. 10.1016/S0140-6736(09)60746-7 19560604

[bib3] Office of National Drug Control PolicyThe Economic Costs of Drug Abuse in the United States, 1992–2002. Executive Office of the President 2004 Contract No: (Publication No. 207303).

[bib4] Belin D, Belin-Rauscent A, Everitt BJ, Dalley JW.. In search of predictive endophenotypes in addiction: insights from preclinical research. Genes Brain Behav 2016; 15: 74–88. 26482647 10.1111/gbb.12265

[bib5] Kendler KS, Chen X, Dick D, Maes H, Gillespie N, Neale MC et al. Recent advances in the genetic epidemiology and molecular genetics of substance use disorders. Nat Neurosci 2012; 15: 181–189. 22281715 10.1038/nn.3018 PMC3297622

[bib6] Stone AL, Becker LG, Huber AM, Catalano RF.. Review of risk and protective factors of substance use and problem use in emerging adulthood. Addict Behav 2012; 37: 747–775. 22445418 10.1016/j.addbeh.2012.02.014

[bib7] Jaenisch R, Bird A.. Epigenetic regulation of gene expression: how the genome integrates intrinsic and environmental signals. Nat Genet 2003; 33: 245–254. 12610534 10.1038/ng1089

[bib8] Jones MJ, Fejes AP, Kobor MS.. DNA methylation, genotype and gene expression: who is driving and who is along for the ride? Genome Biol 2013; 14: 126. 23899167 10.1186/gb-2013-14-7-126 PMC4054606

[bib9] Gaunt TR, Shihab HA, Hemani G, Min JL, Woodward G, Lyttleton O et al. Systematic identification of genetic influences on methylation across the human life course. Genome Biol 2016; 17: 1. 27036880 10.1186/s13059-016-0926-z PMC4818469

[bib10] Joubert BR, Håberg SE, Nilsen RM, Wang X, Vollset SE, Murphy SK et al. 450 K epigenome-wide scan identifies differential DNA methylation in newborns related to maternal smoking during pregnancy. Env Health Perspect 2012; 120: 1425. 22851337 10.1289/ehp.1205412 PMC3491949

[bib11] Richmond RC, Simpkin AJ, Woodward G, Gaunt TR, Lyttleton O, McArdle WL et al. Prenatal exposure to maternal smoking and offspring DNA methylation across the lifecourse: findings from the Avon Longitudinal Study of Parents and Children (ALSPAC). Hum Mol Genet 2015; 24: 2201–2217. 25552657 10.1093/hmg/ddu739 PMC4380069

[bib12] Cecil CM, Walton E, Viding E.. DNA methylation, substance use and addiction: a systematic review of recent animal and human research from a developmental perspective. Curr Addict Rep 2015; 2: 331–346.

[bib13] Wong CC, Mill J, Fernandes C.. Drugs and addiction: an introduction to epigenetics. Addiction 2011; 106: 480–489. 21205049 10.1111/j.1360-0443.2010.03321.x

[bib14] Nestler EJ.. Epigenetic mechanisms of drug addiction. Neuropharmacology 2014; 76: 259–268. 23643695 10.1016/j.neuropharm.2013.04.004 PMC3766384

[bib15] Jung Y, Hsieh LS, Lee AM, Zhou Z, Coman D, Heath CJ et al. An epigenetic mechanism mediates developmental nicotine effects on neuronal structure and behavior. Nat Neurosci 2016; 19: 905–914. 27239938 10.1038/nn.4315 PMC4925298

[bib16] Harlaar N, Hutchison KE.. Alcohol and the methylome: design and analysis considerations for research using human samples. Drug Alcohol Depend 2013; 133: 305–316. 23968814 10.1016/j.drugalcdep.2013.07.026

[bib17] Cecil CAM, Walton E, Viding E.. Epigenetics of addiction: current knowledge, challenges and future directions. J Stud Alcohol Drugs 2016; 77: 688–691. 27588525 10.15288/jsad.2016.77.688

[bib18] Numata S, Ye T, Hyde TM, Guitart-Navarro X, Tao R, Wininger M et al. DNA methylation signatures in development and aging of the human prefrontal cortex. Am J Hum Genet 2012; 90: 260–272. 22305529 10.1016/j.ajhg.2011.12.020 PMC3276664

[bib19] Hicks BM, Iacono WG, McGue M.. Index of the transmissible common liability to addiction: heritability and prospective associations with substance abuse and related outcomes. Drug Alcohol Depend 2012; 123: S18–S23. 22245078 10.1016/j.drugalcdep.2011.12.017 PMC3330176

[bib20] Barker ED, Maughan B.. Differentiating early-onset persistent versus childhood-limited conduct problem youth. Am J Psychiatry 2009; 166: 900–908. 19570930 10.1176/appi.ajp.2009.08121770

[bib21] Fraser A, Macdonald-Wallis C, Tilling K, Boyd A, Golding J, Davey Smith G et al. Cohort profile: the Avon Longitudinal Study of Parents and Children: ALSPAC mothers cohort. Int J Epidemiol 2013; 42: 97–110. 22507742 10.1093/ije/dys066 PMC3600619

[bib22] Boyd A, Golding J, Macleod J, Lawlor DA, Fraser A, Henderson J et al. Cohort Profile: the 'children of the 90 s'—the index offspring of the Avon Longitudinal Study of Parents and Children. Int J Epidemiol 2013; 42: 111–127. 22507743 10.1093/ije/dys064 PMC3600618

[bib23] Saunders JB, Aasland OG, Babor TF, de la Fuente JR, Grant M. Development of the alcohol use disorders identification test (AUDIT): WHO collaborative project on early detection of persons with harmful alcohol consumption—II. Addiction 1993; 88: 791–804. 8329970 10.1111/j.1360-0443.1993.tb02093.x

[bib24] Pidsley R, Y Wong CC, Volta M, Lunnon K, Mill J, Schalkwyk LC. A data-driven approach to preprocessing Illumina 450 K methylation array data. BMC Genom 2013; 14: 293. 10.1186/1471-2164-14-293 PMC3769145 23631413

[bib25] Johnson WE, Li C, Rabinovic A. Adjusting batch effects in microarray expression data using empirical Bayes methods. Biostatistics 2007; 8: 118–127. 16632515 10.1093/biostatistics/kxj037

[bib26] Chen YA, Lemire M, Choufani S, Butcher DT, Grafodatskaya D, Zanke BW et al. Discovery of cross-reactive probes and polymorphic CpGs in the Illumina Infinium HumanMethylation450 microarray. Epigenetics 2013; 8: 203–209. 23314698 10.4161/epi.23470 PMC3592906

[bib27] Price ME, Cotton AM, Lam LL, Farre P, Emberly E, Brown CJ et al. Additional annotation enhances potential for biologically-relevant analysis of the Illumina Infinium HumanMethylation450 BeadChip array. Epigenet Chromatin 2013; 6: 4. 10.1186/1756-8935-6-4 PMC3740789 23452981

[bib28] Cecil CAM, Lysenko L, Jaffee SR, Relton CL, Mill J, Barker ED. Environmental risk, oxytocin receptor gene (OXTR) methylation and youth callous-unemotional traits: a 13-year longitudinal study. Mol Psychiatry 2014; 19: 1071–1077. 25199917 10.1038/mp.2014.95 PMC4231290

[bib29] R Core TeamR: A Language and Environment for Statistical Computing. R Foundation for Statistical Computing: Vienna, Austria, 2015.

[bib30] Muthen LK, Muthen BO. MPLUS User's Guide, 1998–2010, 6th edn. Muthen & Muthen: Los Angeles, CA, USA, 2011.

[bib31] Houseman EA, Accomando WP, Koestler DC, Christensen BC, Marsit CJ, Nelson HH et al. DNA methylation arrays as surrogate measures of cell mixture distribution. BMC Bioinform 2012; 13: 86. 10.1186/1471-2105-13-86 PMC3532182 22568884

[bib32] Wang D, Yan L, Hu Q, Sucheston LE, Higgins MJ, Ambrosone CB et al. IMA: an R package for high-throughput analysis of Illumina's 450 K Infinium methylation data. Bioinformatics 2012; 28: 729–730. 22253290 10.1093/bioinformatics/bts013 PMC3289916

[bib33] Kent WJ, Sugnet CW, Furey TS, Roskin KM, Pringle TH, Zahler AM et al. The human genome browser at UCSC. Genome Res 2002; 12: 996–1006. 12045153 10.1101/gr.229102 PMC186604

[bib34] Pedersen BS, Schwartz DA, Yang IV, Kechris KJ. Comb-p: software for combining, analyzing, grouping and correcting spatially correlated *P*-values. Bioinformatics 2012; 28: 2986–2988. 22954632 10.1093/bioinformatics/bts545 PMC3496335

[bib35] Shah S, Bonder MJ, Marioni RE, Zhu Z, McRae AF, Zhernakova A et al. Improving phenotypic prediction by combining genetic and epigenetic associations. Am J Hum Genet 2015; 97: 75–85. 26119815 10.1016/j.ajhg.2015.05.014 PMC4572498

[bib36] Hu Lt, Bentler PM. Cutoff criteria for fit indexes in covariance structure analysis: conventional criteria versus new alternatives. Struct Equ Model 1999; 6: 1–55.

[bib37] Liu Y, Lv K, Li Z, Yu AC, Chen J, Teng J. *PACSIN1*, a Tau-interacting protein, regulates axonal elongation and branching by facilitating microtubule instability. J Biol Chem 2012; 287: 39911–39924. 23035120 10.1074/jbc.M112.403451 PMC3501072

[bib38] Pérez-Otaño I, Luján R, Tavalin SJ, Plomann M, Modregger J, Liu X-B et al. Endocytosis and synaptic removal of NR3A-containing NMDA receptors by *PACSIN1*/syndapin1. Nat Neurosci 2006; 9: 611–621. 16617342 10.1038/nn1680 PMC1892311

[bib39] Wills MK, Jones N. Teaching an old dogma new tricks: twenty years of Shc adaptor signalling. Biochem J 2012; 447: 1–16. 22970934 10.1042/BJ20120769

[bib40] Huang EJ, Reichardt LF. Trk receptors: roles in neuronal signal transduction. Annu Rev Biochem 2003; 72: 609–642. 12676795 10.1146/annurev.biochem.72.121801.161629

[bib41] Consortium G. The genotype-tissue expression (GTEx) pilot analysis: multitissue gene regulation in humans. Science 2015; 348: 648–660. 25954001 10.1126/science.1262110 PMC4547484

[bib42] Petryszak R, Keays M, Tang YA, Fonseca NA, Barrera E, Burdett T et al. Expression Atlas update—an integrated database of gene and protein expression in humans, animals and plants. Nucleic Acid Res 2015; 44: D746–D752. 26481351 10.1093/nar/gkv1045 PMC4702781

[bib43] Colantuoni C, Lipska BK, Ye T, Hyde TM, Tao R, Leek JT et al. Temporal dynamics and genetic control of transcription in the human prefrontal cortex. Nature 2011; 478: 519–523. 22031444 10.1038/nature10524 PMC3510670

[bib44] Philibert R, Hollenbeck N, Andersen E, Osborn T, Gerrard M, Gibbons FX et al. A quantitative epigenetic approach for the assessment of cigarette consumption. Front Psychol 2015; 6: 656. 26082730 10.3389/fpsyg.2015.00656 PMC4451580

[bib45] Schael S, Nüchel J, Müller S, Petermann P, Kormann J, Pérez-Otaño I et al. Casein kinase 2 phosphorylation of protein kinase C and casein kinase 2 substrate in neurons (PACSIN) 1 protein regulates neuronal spine formation. J Biol Chem 2013; 288: 9303–9312. 23420842 10.1074/jbc.M113.461293 PMC3611001

[bib46] Volkow N, Baler R. Addiction science: uncovering neurobiological complexity. Neuropharmacology 2014; 76: 235–249. 23688927 10.1016/j.neuropharm.2013.05.007 PMC3818510

[bib47] Rijlaarsdam J, Cecil CAM, Walton E, Mesirow MSC, Relton CL, Gaunt TR et al. Prenatal unhealthy diet, insulin-like growth factor 2 gene (*IGF2* methylation, and attention deficit hyperactivity disorder symptoms in youth with early-onset conduct problems. J Child Psychol Psychiatry 2016; doi: 10.1111/jcpp.12589 (e-pub ahead of print). 10.1111/jcpp.12589 PMC5161647 27535767

[bib48] Walton E, Pingault J-B, Cecil C, Gaunt T, Relton C, Mill J et al. Epigenetic profiling of ADHD symptoms trajectories: a prospective, methylome-wide study. Mol Psychiatry 2016; doi:10.1038/mp.2016.85 (e-pub ahead of print). 10.1038/mp.2016.85 PMC5014094 27217153

[bib49] Barker DJ. The origins of the developmental origins theory. J Intern Med 2007; 261: 412–417. 17444880 10.1111/j.1365-2796.2007.01809.x

[bib50] Heijmans BT, Mill J. Commentary: the seven plagues of epigenetic epidemiology. Int J Epidemiol 2012; 41: 74–78. 22269254 10.1093/ije/dyr225 PMC3304528

[bib51] Pingault J-B, Cecil C, Murray J, Munafò MR, Viding E. Causal inference in psychopathology: a systematic review of Mendelian randomisation studies aiming to identify environmental risk factors for psychopathology. Psychopathol Rev 2016; Available at: http://dx.doi.org/10.5127/pr.038115 (accessed on 21 February 2016).

[bib52] Relton CL, Davey Smith G. Two-step epigenetic Mendelian randomization: a strategy for establishing the causal role of epigenetic processes in pathways to disease. Int J Epidemiol 2012; 41: 161–176. 22422451 10.1093/ije/dyr233 PMC3304531

[bib53] Dwyer JB, McQuown SC, Leslie FM. The dynamic effects of nicotine on the developing brain. Pharmacol Ther 2009; 122: 125–139. 19268688 10.1016/j.pharmthera.2009.02.003 PMC2746456

[bib54] Muhammad A, Mychasiuk R, Nakahashi A, Hossain SR, Gibb R, Kolb B. Prenatal nicotine exposure alters neuroanatomical organization of the developing brain. Synapse 2012; 66: 950–954. 22837140 10.1002/syn.21589

